# Engaging Youth in the Bipolar Youth Action Project: Community-Based Participatory Research

**DOI:** 10.2196/19475

**Published:** 2020-09-10

**Authors:** Laura Lapadat, Anusha Balram, Joanna Cheek, Eugenia Canas, Andrea Paquette, Bipolar Youth Action Group, Erin E Michalak

**Affiliations:** 1 Department of Psychiatry University of British Columbia Vancouver, BC Canada; 2 Faculty of Information and Media Studies Western University London, ON Canada; 3 Stigma-Free Society Vancouver, BC Canada

**Keywords:** community-based participatory research, bipolar disorder, adolescent, young adult, youth, participatory research

## Abstract

**Background:**

We describe the methodological dimensions of community-based participatory research through a description of study design, youth engagement, and methods/processes in the cocreation of knowledge within a Canadian study, the Bipolar Youth Action Project. This collaborative partnership—carried out by a team composed of academic, community, and youth partners—was designed to investigate self-management and wellness strategies for young adults living with bipolar disorder.

**Objective:**

The aim is to describe the opportunities and challenges of this collaboration and to reflect upon the process of involving youth with bipolar disorder in health research that concerns them, and share lessons learned.

**Methods:**

The project was conducted in multiple phases over 2 years: (1) grant-writing, with youth contributing to the process; (2) recruitment, in which 12 youth were selected and trained to help shape and conduct two research forums; (3) the first research forum, where more youth were consulted about the strategies they apply to stay well (self-management strategies); (4) data analysis of Forum I findings; (5) research Forum II, which consulted youth with bipolar disorder about knowledge translation of Forum I findings; and (6) data analysis of Forum II findings. Youth peer researchers with bipolar disorder were involved in a significant capacity at every stage in the process.

**Results:**

Of the initial 12 youth peer researchers, 7 remained on the project from the recruitment phase until the project ended. They collaborated in the creation of two youth research forums that consulted youth with bipolar disorder on their self-management strategies.

**Conclusions:**

This article shares what was learned from the process of partnering with youth with bipolar disorder in a community-based participatory research study.

## Introduction

Bipolar disorder is a type of mood disorder characterized by periods of depressed and elevated (manic or hypomanic) mood states, with corresponding changes in thinking and behavior [1]. The typical onset of bipolar disorder occurs in late adolescence to early adulthood [2]. According to the World Health Organization, the condition is the 6th leading cause of disability among people aged 0-59 years in higher-income countries, and 8th in lower-income countries [3]. Globally, bipolar disorder is the 4th greatest cause of disability-adjusted life years in people aged 10-24 years [4]. Youth, therefore, are a key target group for early intervention and support, particularly given that interventions for bipolar disorder may be more effective for younger adults than for their older counterparts [5,6].

Fostering self-management strategies—that is, the plans and routines that a person with bipolar disorder uses to promote health and quality of life [7]—is viewed as a key element of ensuring optimal health in people with the condition. A solid body of evidence now exists on self-management in adults with bipolar disorder [7-11]. However, much less is known about effective self-management for bipolar disorder in youth populations [10].

### Community-Based Participatory Research

The research methods of the Bipolar Youth Action Project were guided by the Collaborative RESearch Team to study psychosocial issues in Bipolar Disorder (CREST.BD) [12], a Canada-based network dedicated to collaborative research and knowledge translation in bipolar disorder. The CREST.BD network specializes in community-based participatory research, a participatory research approach in which academic researchers and community members work in partnership [13]. Community-based participatory research aims to shape research around community priorities, emphasizing knowledge generation that contributes to the community and social change [13-15]. Community-based participatory research can be viewed as a “philosophy of engagement” [16] rather than a discrete method per se; instead, diverse methodological approaches can be applied within the frameworks of the research method. The CREST.BD team, who are informed by a decade of research and integrated knowledge translation, developed a specific model of community-based participatory research for bipolar disorder [17]. The model builds on the strengths of the community of people with bipolar disorder and has been successfully applied across diverse projects in populations of adults with bipolar disorder [9,17-19].

A logical progression in CREST.BD’s program of research was to explore the application of community-based participatory research approaches in youth and young adults living with bipolar disorder. Community-based participatory research has long been viewed as an effective approach for working with underserved populations, gaining increasing support for its use over the past twenty years [20-23]. Seldom heard populations, including populations of youth [24], voice a need for innovative approaches to address issues of social, contextual, language, and cultural factors faced in mental health treatment systems. For youth facing mental health challenges, community-based participatory research approaches hold potential to amplify well-being and personal strengths [25], increase access to mental health services and information [26], and enhance methodological rigor and implementation of research findings [20,24].

Diverse and international youth populations have now been engaged in community-based participatory research projects [24]. In the mental health arena, these approaches have been used to advance knowledge on substance use in youth [27]; suicidality in American Indigenous youth [21]; trauma, psychiatric issues and educational and behavioral outcomes in Cambodian American youth [28], and bullying in elementary-aged youth [29]. The potential assets of community-based participatory research approaches in youth mental health research are clear. However, there are particular challenges in participatory research with youth, including difficulties concerning power differentials [30], problems maintaining engagement [31], concerns of disclosure and anonymity [32], and differing research goals between academic and peer research teams [30].

In summary, we know that self-management strategies are critical for health and quality of life in people with bipolar disorder, but a gap exists in self-management research specifically addressing youth with the condition. A specific model for community-based participatory research in adults with bipolar disorder has been developed, but little is yet known about the application of this approach in youth. This article traces the lifespan of a 2-year community-based participatory research-informed study engaging youth with bipolar disorder as peer researchers. We describe how youths’ roles as peer researchers presented both opportunities and challenges to more traditional research and knowledge translation activities. We also outline the lessons learned from this process.

## Methods

### Project Aims and Overarching Design

The Bipolar Youth Action Project was a 2-year youth-driven research project with a primary aim of building knowledge on (1) effective self-management strategies for youth with bipolar disorder, and (2) preferred knowledge translation methods for sharing this knowledge with other youth with bipolar disorder and their supporters. A secondary aim was to develop the knowledge base on the application of community-based participatory research in youth living with bipolar disorder. Youth with bipolar disorder were integral to each stage of the Bipolar Youth Action Project, as funding coapplicants, peer researchers, research participants, and knowledge brokers. Previous Bipolar Youth Action Project publications have described the project’s specific methods [33] and provided a qualitative analysis of youth preferences for knowledge translation methods for online health information [34]. Here, we take a deeper dive into our secondary aim, which is to describe and share reflections on the community-based participatory research approaches we undertook in the Bipolar Youth Action Project. In the following sections, we describe the specific methods we undertook across the various study phases.

### Prefunding Phase

The Bipolar Youth Action Project was funded by the Vancouver Foundation, a British Columbia-based funding agency dedicated to supporting community-focused research. The funding application was coproduced in equal partnership by two organizations: the Bipolar Disorder Society of British Columbia (BDSBC; now Vancouver BC-based Stigma-Free Society), an organization providing social support and services for people living with bipolar disorder based in Victoria, British Columbia, and CREST.BD, headquartered in Vancouver. In order to generate pilot data for the funding application, the Executive Director of BDSBC conducted a focus group with five youth with bipolar disorder who were associated with the BDSBC. The youths were recruited through announcements at BDSBC’s support groups and via advertisements within the community and in a local newspaper. Youth applicants were required to submit a resume and cover letter and were interviewed by the Executive Director of the BDSBC and by the project’s Principal Investigator. The inclusion criterion for eligibility was self-report of a health care provider diagnosis of bipolar disorder (type I, II, or not otherwise specified). Two of the youth who participated in the focus group self-selected to serve as coapplicants on the funding application and collaborated on the identification of project methods.

### Phase 1: Team Establishment and Capacity Building

#### Team Establishment

Central to the Bipolar Youth Action Project was the establishment of the Youth Action Group, a group of young adults aged 20 to 25 years living with bipolar disorder, who served as peer researchers for the duration of the project. The BDSBC recruited 12 youth by advertising the project within their community network and interviewing prospective members, with the expectation that there would be some attrition over the study’s 2-year span. The recruitment process and inclusion criteria were the same as in the prefunding phase, with the addendum that members of the Youth Action Group must also be able to complete a 2.5-hour research ethics course (the Tri-Council Policy Statement Course on Research Ethics). Two “Co-Leads” self-selected to take on the additional responsibilities of governance and leadership of the Youth Action Group. The wider project team consisted of an academic researcher specializing in community-based participatory research in bipolar disorder, the Executive Director of the BDSBC, a specialist from an organization dedicated to youth engagement, health care providers (two psychiatrists who were coapplicants on the funding application and one mental health counselor), and a research coordinator.

#### Capacity Building

A series of four foundational training sessions were conducted with Youth Action Group members to build capacity. The sessions were focused on: the principles and implementation of community-based participatory research; qualitative, quantitative and graphic facilitation methods; research ethics and knowledge translation; and providing a grounding in CREST.BD’s previous research exploring self-management in adults with bipolar disorder (see Table 1).

Graphic facilitation, or graphic recording, is a process of illustrating themes and ideas shared during discussions using a combination of text and imagery, typically on a whiteboard or large sheet of paper [35]. The rationale for training Youth Action Group members in graphic facilitation was twofold. First, it was a means of knowledge translation of findings from CREST.BD’s research into adult self-management, as group members were tasked with visualizing previous findings as the academic team presented them. Second, introducing graphic facilitation to group members provided training in a knowledge translation method that could be utilized at the research forums.

**Table 1:** Phase I research training events.

Training Day	Purpose	Description
Research training day	YAG^a^ team-building, education about CBPR, qualitative methods	Full day in-person event
Graphic facilitation day	Educate YAG about self-management and graphic facilitation methods	Full day in-person event
TCPS2 CORE course^b^	Train all YAG members in research ethics	Online research ethics course for researchers and research staff
CBPR^c^ webinar	Refresh YAG knowledge of CBPR and qualitative research methods	Web-based presentation at the end of Phase 1

^a^YAG: Youth Action Group.

^b^The Tri-Council Policy Statement Tutorial Course in Research Ethics (Government of Canada Panel on Research Ethics, 2016).

^c^CBPR: community-based participatory research.

### Phase 2: Forum I

Once the training phase was complete, team members co-designed and delivered the first of two “Youth Research Forums,” hosted at an event center chosen by the Youth Action Group. The primary research goal of Forum I was to yield new knowledge on self-management of bipolar disorder in youth. A secondary aim, determined by the Youth Action Group, was to share knowledge of bipolar disorder self-management and stigma in the form of group-designed workshops and presentations. In order to include the perspectives of youth in high school, participants in Forum I could be aged 16-25 years, and self-identified as living with bipolar disorder I, II, or not otherwise specified. Recruitment was undertaken jointly by the BDSBC, who reached out within its network, and Youth Action Group members, who distributed posters and volunteered at mental health awareness events.

**Figure 1:**
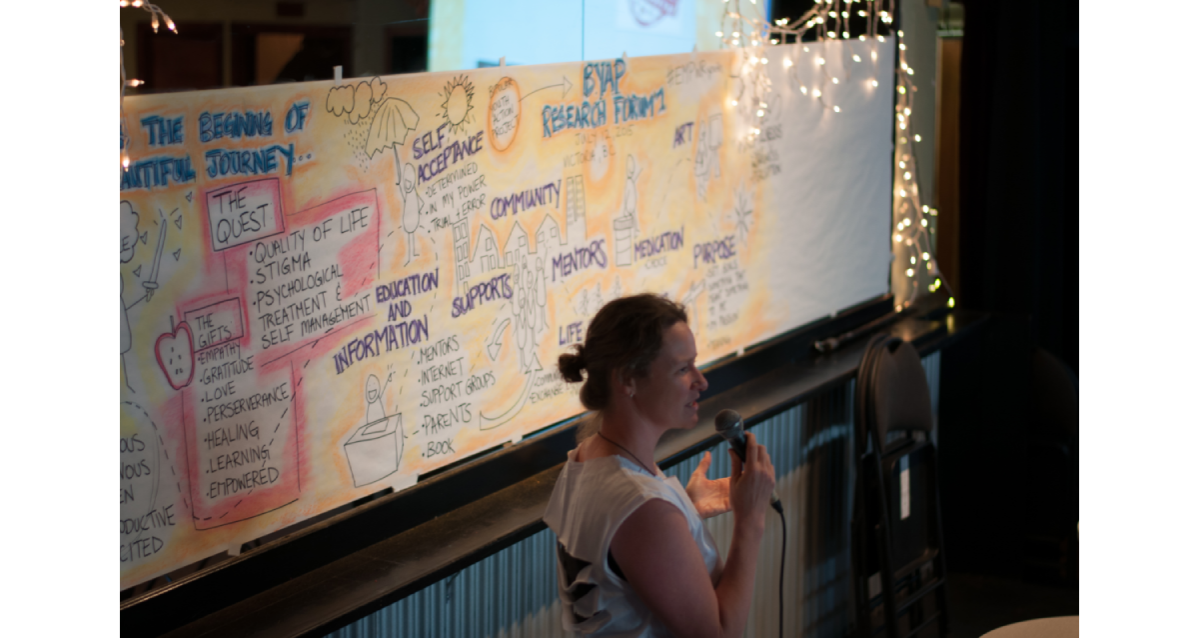
Erin Stewart Elliott, the project’s graphic facilitator, presents her illustrations of the day’s themes at Forum I.

At the opening of the day, Youth Action Group members delivered presentations and workshops to forum attendees, followed by a group-led mindfulness activity. Afterward, academic research team members with experience in qualitative methods moderated four 90-minute focus groups to discuss self-management strategies used by youth to stay well. Focus groups were digitally recorded, with Youth Action Group members acting as notetakers. Throughout Forum I, a graphic facilitation specialist visualized themes from the Youth Action Group presentations on a large sheet of paper hung on the wall (Figure 1).

A private area was available for participants to retreat to in case of distress, and health care providers (one female, one male) were available on-site to provide support as needed. One forum participant opted to bring a parental supporter with them to the event (but the supporter did not participate in data collection or discussions).

### Phase 3: Forum I Analysis

A Youth Action Group member transcribed the focus group audio recordings verbatim and ensured all identifying information was removed. A coding framework was collaboratively developed with the Youth Action Group member and two academic researchers; the Youth Action Group member also participated in the thematic analysis (findings reported elsewhere) [33]. The same member created a summary of findings to present to the rest of the group during Forum II planning.

### Phase 4: Forum II

The primary goal of Forum II was to share the results of Forum I with participants and to consult on suggestions for knowledge translation and dissemination of Forum I findings. Inclusion criteria for Forum II were the same; both prior Forum I participants and new youth participants were welcome to join. Youth Action Group members shared the results of Forum I using presentations, arts-based methods, and group activities. As at the previous Forum, a graphic facilitator illustrated themes discussed and shared throughout the day on a large wall-mounted sheet of paper.

After the presentations and workshops, youth participated in a World Café to discuss avenues for knowledge translation. World Café is a method of structured conversation in which participants move between multiple tables, each with a designated topic of discussion. Academic researchers served as facilitators at each of three tables, and one table was facilitated by the Youth Action Group Co-Leads. Other members joined discussions as participants. The conversations at each table were recorded.

### Phase 5: Forum II Analysis

World Café audio recordings were analyzed by a Youth Action Group member and another researcher. Detailed descriptive notes were written, eliminating identifying information, with tallies tracked of concrete suggestions made for knowledge translation.

### Ethical Considerations

The University of British Columbia Behavioural Research Ethics Board and the Island Health Research Ethics Board granted ethics approval for the Bipolar Youth Action Project. Youth aged 19 years or above provided written consent to participate in the study; youth aged 16 to 18 years provided both their written assent and written consent of a parent or guardian. Consent packages were written in plain language to a Flesch-Kincaid reading level of grade 8 and included discussion around the permanency of media (photos and video) produced as products of the study. For data-gathering, consent to record audio was a prerequisite for participation in both forums, but participants, including the Youth Action Group, could decline consent for photography and videotaping.

### Compensation Considerations

Youth Action Group members were compensated at a rate of USD42 (CAD50) per monthly meeting; Co-Leads were compensated at a rate of USD55 (CAD65) per meeting. One member was employed on contract to conduct the transcription, and support the coding and data analysis, of Forum I results, and the transcription and initial analysis of Forum II results. Youth Action Group members received reimbursement for childcare and travel expenses, and meals were provided at all meetings.

## Results

### Recruitment and Retention

At the outset of the Bipolar Youth Action Project, 12 young adults (aged 20-25; 10 females and 2 males) living with bipolar disorder were recruited to the Youth Action Group. All members were Caucasian and resided within urban areas. One group member was a parent. At the end of the 2-year project, 7 of the 12 original members (58.3%; 6 females and 1 male) remained engaged, a notable retention rate for a youth project of this length. Three members of the Youth Action Group (all female) discontinued participation in the because of illness relapse or reported difficulties balancing project responsibilities with university and work commitments. 2 additional youth (1 female, 1 male) discontinued without citing a reason, and the team was unable to reach them for follow-up. Of note, all attrition of Youth Action Group members was before the final training session; from this point until the project end, the seven members remained engaged.

### Youth Roles and Capacity-Building

Youth Action Group members’ participation in the Bipolar Youth Action Project was hybrid in nature. First, the members acted as paid peer researchers. Their role included helping to inform and guide the research direction, organize and present at the research forums, and disseminate study results. The members attended monthly meetings, participated in training sessions, and maintained continuous communication with the academic team via email, and with one another via Facebook. Throughout the project, members took on project roles and built capacity in areas that interested them, including meeting agenda cocreation (n=2); event planning (n=7); social media management and outreach (n=4); creation of infographics and video presentations (n=7); public speaking (n=7); data analysis (n=1); event cohosting (n=4); and knowledge translation of self-management strategies into engaging presentations (n=7).

Second, Youth Action Group members acted as research participants themselves. At Forum II, the group explicitly contributed knowledge about how best to share youth bipolar disorder self-management strategies with the wider community, and their experiences with participating in the project as peer researchers was an important source of knowledge about youth community-based participatory research engagement.

Facilitating Youth Action Group members’ contributions and roles throughout the project required a flexible approach, with the community partner and academic team adjusting their work schedules to accommodate work and school schedules. Attention was paid to considering how to develop rapport effectively, convey information in accessible and engaging terms, assign appropriate quantities of work, and aid the youth peer researchers in taking ownership of the project. The group members worked most effectively with clear deadlines and consistent email communications.

Training events contributed to cohesion and trust-building within the overall team, which helped to model, build, and maintain effective working relationships and retain participants. Although significant staff time and explicit funding were set aside for Youth Action Group training during the eight months of phase I, in practice, training was ongoing throughout the project on a less formal basis. Given the length of the study, the members would likely have benefitted from refresher training on concepts learned earlier in the project.

Youth became more secure in their roles and in taking ownership of the Bipolar Youth Action Project as it progressed, demonstrating a greater willingness to take on leadership roles after they had been delineated or modeled by the community partner and academic researchers. For Forum I, five of the seven Youth Action Group members elected to give workshops on specific topics related to mental health. During this process, the academic team supported the members in selecting topics and encouraging them to develop their ideas. The chosen topics were (1) living with mental health stigma, (2) creating a personal mindfulness toolbox, and (3) “Leading Extraordinary Lives” (Table 2). Although the members had been trained in graphic facilitation, they chose not to partake in this at Forum I.

Youth Action Group members were much more confident assuming leadership for the second Forum and wrote and delivered workshops with minimal involvement of the academic researchers. Of the 7 Youth Action Group members, six were split into pairs, and each chose to present on themes from Forum I. The chosen topics were: (1) health and fitness, (2) in-the-moment strategies for managing shifts in mood, and (3) developing support networks (Table 2). As with Forum I, Youth Action Group members demonstrated creativity and passion towards the topics chosen; however, they were notably more confident in the development and execution of their ideas for Forum II and required less input and encouragement from the academic team. At Forum II, Youth Action Group members again elected not to partake in graphic facilitation of presentations and workshops, although they did create some writings and illustrations with other participants during the introduction phase of the day.

**Table 2:** Youth Action Group­–led and designed workshops.

Workshop topic		Description
**Forum I**		
	Stigma	One member and the community partner delivered a presentation explaining the stigma of bipolar disorder and how to live with it.
	Mindfulness	Two members introduced the topic of mindfulness and led participants in creating personal mindfulness toolboxes.
	Leading Extraordinary Lives	Two members presented their personal health stories, with the underlying message that it is possible to live an extraordinary life with bipolar disorder.
**Forum II**		
	Health and fitness	Two members shared their personal health stories as a means of knowledge translation of Forum I findings of self-management through diet and fitness.
	In-the-moment strategies	Two members used props to demonstrate strategies for self-management while amid a depressive or manic/hypomanic episode, as a means of knowledge translation of Forum I findings.

### Evolving Communication

A common workflow was established reflexively over time, based on group feedback and the academic team’s observations of how engagement varied as a function of project stage, volume of communication, and level of structure provided by the academic team. Email communications served as an important supplement to monthly in-person meetings (especially as the project was being conducted from Vancouver Island and the city of Vancouver), and the academic research team noted that the greater the number of email communications, the more engaged the Youth Action Group appeared to be. Often, deadlines would be agreed upon during in-person meetings, but friendly check-ins during the monthly helped members to meet these deadlines and to be open about any roadblocks or challenges they faced.

### Data Analysis

As noted elsewhere [33], Youth Action Group members expressed that conveying the forum findings in peer-reviewed papers was a priority for them. One member stated an interest in becoming more heavily involved in data collection and analysis. This individual was hired as a contract research assistant to transcribe the Forum I focus group proceedings, and then to take the lead as a peer researcher in the analysis of the focus group findings. The process of involving this peer researcher was mutually beneficial. It helped to develop their capacity in their area of interest, and their lived experience lens was valuable in selecting themes likely to hold relevance towards youth living with bipolar disorder.

## Discussion

### Maintaining Engagement

A particular challenge in engaging youth as coproducers of health research is accommodating their fluctuating school and work schedules [31,36]. In the case of the Bipolar Youth Action Project, it was also expected that Youth Action Group members’ active mood episodes could affect retention and participation. Indeed, for these reasons, some group members did leave the project during Phase 1; however, participation remained consistent after that.

The following are several recommendations to maintain youth engagement.

#### Foster Motivation With Open Communication Around Project Goals and Codevelop Goals to Include Participation of All Team Members

The academic research team found it helpful to ground research activities in the context of a grander purpose. It was important to ensure that youth peer researchers understood how specific activities related to the project aims; research activities that seem obvious to academic team members can appear to be nonsequiturs to youth if they are not informed of the reasoning behind them. Concrete actions that the academic team took towards this aim included: creating an open atmosphere where questioning was encouraged; conveying information through means that involved active and creative participation of the peer researchers, such as graphic facilitation; hosting a final training session that re-stated learnings from previous sessions; opening discussions for youth expertise, and maintaining consistent communication.

#### Reassure Youth That Their Input Is Valued and Take Care to Facilitate Their Input

Peer researchers may feel discouraged from contributing to discussions if they feel their input is not valued [37]. Therefore, creating an environment in which peer researchers’ thoughts and opinions are respected helps them to feel their input is legitimate [38]. Community partners can be useful towards this aim, serving as a liaison between academic and peer researchers, and representing the interests of the target population [37]. The familiarity and trust Youth Action Group members felt with the community partner from the project outset aided them in expressing their points of view. In addition, cocreation of meeting agendas with Youth Action Group Co-Leads ensured that youth input would be woven directly into the structure of discussions.

#### Assign Concrete Responsibilities to Serve as a Mechanism for Engagement

In the context of youth-adult partnerships in mental health research, a balance of flexibility with clear expectations has been found beneficial [39]. Indeed, Youth Action Group member engagement peaked before and during research forums, when tasks to be executed were most concrete. Throughout more abstract phases of the Bipolar Youth Action Project, youth peer researchers were less engaged and required more follow-up to ensure they were aware of meeting times and tasks that needed completion. At these times, group members may have felt unsure about what they were meant to do and had had little reason to think of the Bipolar Youth Action Project amid competing priorities. Establishing more concrete responsibilities early in the project, and contextualizing them as vital for the forums, would likely have helped the youth become more engaged in the project in its earlier stages.

#### Prioritize Effective Communication

Disengagement in participatory research has been attributed in part to mismanagement of roles and a lack of clear, open communication [40]. Keeping consistent contact, such as meeting reminders and communication about assigned work, can improve output and engagement [41]. Throughout the Bipolar Youth Action Project, consistent email communication enhanced peer researcher engagement. It was also important to convey information to members in terms that were accessible and considerate of their condition. The sharing of prior research findings during a training event using graphic facilitation methods provided grounding in CREST.BD’s earlier work in bipolar disorder self-management in an engaging and self-relevant manner.

In CREST.BD’s previous work, the pace of research has, at times, been seen as slow and frustrating by peer researchers [42]. A strategy of pre-emptive sharing of “research snapshots” with the group before extensive analysis of the data has been effective in past community-based participatory research projects conducted by CREST.BD [42]. This strategy was utilized between the first and second forum, at meetings, and through email communications. Early iterations of papers were shared with Youth Action Group members before submission, both for their input and approval, as well as to assure them that their efforts were seeing fruition.

### Capacity-Building Over Time

#### Leadership

In CREST.BD’s prior research, tensions have been experienced in terms of meeting projects’ funded research goals in addition to peer researcher or community goals [42]. In the Bipolar Youth Action Project, Youth Action Group members tended to focus on goals relating to advocacy and immediate action, whereas the academic researchers were oriented towards research and knowledge translation goals. Negotiating these diverse orientations required a reflexive process of establishing spheres of leadership, supporting Bipolar Youth Action Project’s peer researchers to put project outputs into practice and make an immediate impact during forums. At the same time, academic research team members focused more on enacting research goals. A recent publication in youth participatory research posits that, rather than exercising paternalistic direction over youth peer researchers in the interest of promoting research goals, it can be beneficial to consider youth goals “in parallel” to those of academic researchers: distinct, yet proceeding in the same direction [30].

#### Power Inequities

Unequal power differentials are a perennial challenge within participatory research [20,26,30,43], with academic researchers holding an advantage of greater scholarly knowledge, research experience, and status within research projects [44]. Peer researchers hold power and expertise in their own right, through lived experience [39,45], but this power may not be broadly acknowledged [44]. In projects like the Bipolar Youth Action Project, with younger peer researchers, these power inequities may be amplified. Youth, accustomed to hierarchical contexts at school and work, may find it unnatural to be called upon to collaborate as peers, presenting academic researchers with the task of providing necessary supports to encourage collaboration. Affording Youth Action Group members an arena in which to ask questions, and to research their ideas in a working context, was therefore viewed as an important opportunity for helping them to build capacity.

### Time Considerations

Commentators in participatory research have cautioned that inadequate time for involvement by peer researchers can render their participation superficial. Researchers advocate for thoughtful training with time built in for flexibility and delays [40,46]. The “publish or perish” mentality can compel academic researchers to leave insufficient time for the unfolding of participatory research processes, and this risks rendering the involvement of community members tokenistic [40]. The 2-year timeline of the Bipolar Youth Action Project provided the academic team adequate time to reflect upon and adjust and communicate expectations of Youth Action Group members and allowed the members to build capacity over time. In working with youth, who may feel less comfortable assuming responsibility and stating their perspectives openly, longer timelines can provide ample space for the gradual building of capacity and confidence and assumption of responsibility.

#### Settle Collectively on a Workflow That Suits Both Parties and Provide Adequate Structure for Youth Contributions.

Establishing principles of work is foundational to creating positive working relationships within a peer research group [47], which in turn can equalize and enhance patient-led research [45]. The timeline of the Bipolar Youth Action Project allowed consideration towards developing a common workflow that suited the needs of the Youth Action Group. When group members lacked clarity, they were not confident in taking action. Therefore, the process that emerged was one of continuous communication between meetings through email, frequent check-ins, specified deliverables, and clear deadlines. The authors encourage academic researchers undertaking community-based participatory research with populations of youth to determine a workflow that suits youth peer researchers collectively and to be willing to commit extra time and resources to form structure, communication, and scaffolding to foster youth involvement.

### Compensate Peer Researchers for Their Time and Effort

Within participatory research, failing to provide adequate compensation can lead to disengagement and disempowerment of peer researchers [40]. Compensation creates an environment of reciprocity, in which all participants feel valued [38]. Compensation takes multiple forms, including payment for work, reimbursement for expenses such as transit, provision of meals and snacks, demonstration of respect and appreciation, and public acknowledgment of contributions [38]. Within the Bipolar Youth Action Project, sharing meals at each meeting helped to build trust and mutuality between peer researchers and academic researchers. Monetary compensation reinforced member expertise in the Youth Action Group and demonstrated the value of their contributions.

### Be Sensitive to Ethical Concerns

In the application of community-based participatory research with seldom-heard populations of youth, as in the Bipolar Youth Action Project, a considered approach to ethics is necessary to ensure safety [47]. Most evident is the need to ensure that health professionals are available should distress occur, and to carefully convey sensitive information [40,47].

#### Disclosure and Anonymity

A particular challenge within the Bipolar Youth Action Project was disclosure. In recent years, research approaches have shifted from championing anonymity towards the notion that it can be empowering for participants to choose for themselves whether they would like to remain anonymous [32]. However, bipolar disorder is undeniably stigmatized [48], and this presents a concern for many people with the condition [17]. Most group members were comfortable having their names and photographs shared as a part of the coproduction of materials, but some were not. It was challenging to simultaneously meet the goals of coproduction and empowerment within community-based participatory research with the need to protect confidentiality and when requested, anonymity. In the Bipolar Youth Action Project, this was resolved by only photographing and sharing the names of those who consented to do so, and through ongoing dialogues regarding when and where the Youth Action Group members were comfortable being identified. Of the many materials produced throughout the 2-year project, it was sometimes possible to include all members of the team while omitting their names and or diagnoses from the shared material. Continuous communication with Youth Action Group members was essential to ensuring that they felt empowered and credited when desired, without sacrificing confidentiality and anonymity of other members.

#### Relationships Between Community Organizers and Peer Researchers

Another ethical consideration underscored by this project was the reliance of the participants, often members of stigmatized, seldom heard, and at-risk populations, upon the services provided by research or community organizations involved in a community-based participatory research project. In the case of the Bipolar Youth Action Project, the majority of Youth Action Group members were recruited through their attendance at BDSBC support groups, which they may have depended upon for support and connection to others with bipolar disorder. This reliance on organizations involved in community-based participatory research may represent a form of implicit coercion, in that it could compel participants to remain involved not for the sake of the project, but for the sake of maintaining positive relationships with those providing essential health services to them. With this in mind, we recommend continually reassuring community-based participatory research participants that their relationship to these organizations and services is not contingent on their continued involvement, and that, regardless of the outcome of their participation in the study, the team’s highest priority is that community members are not facing barriers to service access.

### Limitations

There were some limitations to the Bipolar Youth Action Project. All Youth Action Group members were white and resided within an urban area. The study was conducted on the traditional territories of the Songhees and Esquimalt First Nations; however, no participants self-identified as having First Nations heritage, and we were unable to engage with local First Nations as a part of this project to gauge their interest in the research. Only binary gender identities were represented. Only 2 of 12 members were male-identified at study outset; at its conclusion, only 1 of the male-identified members remained. It is unknown if any LGBTQ+ representation was present in the group, as this information was not requested or shared. Our results are not, therefore, easily generalizable to racialized populations, populations with diverse gender and sexual identities, and populations living outside of urban areas.

### Conclusions

This article has described challenges and lessons learned during a community-based participatory research project involving youth with bipolar disorder. As compared with other forms of participatory research, research that involves youth may require additional time, communication, support, and attentiveness to power differentials. Youth motivations may differ from adult and academic researchers, and it is important to codeliver on both youth-identified and academic research priorities. We hope that this article will contribute to the knowledge base on conducting participatory mental health research and aid others in the design of mutually beneficial participatory research projects with youth populations.

## Abbreviations

BDSBC: Bipolar Disorder Society of British Columbia
CREST.BD: Collaborative RESearch Team to study psychosocial issues in BD
